# The perceived utility and scope of ecological momentary assessment and interventions for non-suicidal self-injury: A needs-based dynamic risk perspective

**DOI:** 10.1016/j.invent.2026.101014

**Published:** 2026-10-24

**Authors:** Mirthe Luijsmans, Laurence Claes, Ruth Tatnell, Marlies Houben, Thomas Vaessen, Tom Smeets, Inez Myin-Germeys, Glenn Kiekens

**Affiliations:** a Center for Contextual Psychiatry, Department of Neurosciences, KU Leuven, Belgium; b Department of Medical and Clinical Psychology, Tilburg University, the Netherlands; c Research Unit Clinical Psychology, Faculty of Psychology and Educational Sciences, KU Leuven, Belgium; d Faculty of Medicine and Health Sciences (CAPRI), University of Antwerp, Belgium; e School of Psychology, Deakin University, Burwood, Victoria, Australia; f Department of Psychology, Health & Technology, University of Twente, the Netherlands

**Keywords:** Non-suicidal self-injury, Ecological momentary assessment (EMA), Ecological momentary intervention (EMI), Just-in-time adaptive intervention (JITAI), Qualitative research, Reflexive thematic analysis

## Abstract

**Background:**

Non-suicidal self-injury (NSSI) is characterized by rapid within-person fluctuations in thoughts and urges. Yet, treatments rely on retrospective and static approaches for assessments and interventions. Ecological Momentary Assessment (EMA) and Ecological Momentary Interventions (EMIs) may enable more dynamic support in real time. This study examined the perceived utility and scope of these new digital modalities from the perspectives of treatment-seeking individuals and mental health professionals.

**Methods:**

Semi-structured interviews were conducted with 30 treatment-seeking individuals engaging in NSSI, after completing a four-week EMA period, and with 14 professionals who used EMA in treatment. Interviews explored experiences with EMA and perspectives on EMIs and were analyzed using reflexive thematic analysis.

**Results:**

Three themes were identified. First, EMA and EMIs were perceived as extending therapeutic support by enhancing awareness, reflection, and skill use in daily life. Second, participants emphasized that EMIs must be tailored to changing needs in function of changing risk. This informed the development of a Needs-Based Dynamic Risk and Intervention Model for NSSI, distinguishing needs and interventions through a traffic-light analogy across resilient (i.e., mental health promotion), emerging risk (i.e., vulnerability regulation), and high risk (i.e., behavioral interruption) states. Third, EMIs were viewed as valuable when embedded in blended care and implemented through shared decision-making, balancing system- and user-initiated support.

**Conclusion:**

Integrating EMA and EMIs into treatment has potential to provide needs-tailored support for individuals who self-injure. Future work should focus on empirically validating the proposed dynamic model and co-designing EMI components that address fluctuating needs in daily life.

## Highlights

• A novel Needs-Based Dynamic Risk and Intervention Model for NSSI is introduced.
• Support needs change dynamically across different states of risk.
• Future EMIs should adapt to changing momentary needs and risk.
• EMA and EMIs can bridge the gap between therapy and daily life.
• Digital interventions are best embedded in blended care and shared decision-making.

## 1 Introduction

Non-suicidal self-injury (NSSI), defined as the intentional injury of one's own body tissue without suicidal intent (e.g., cutting or hitting oneself), is a major mental health concern worldwide (International Society for the Study of Self-Injury, 2026). Prevalence rates are high, especially among clinical populations, with up to one in ten adults and nearly half of adolescents receiving mental health treatment reporting NSSI in the past month (Muehlenkamp and Tillotson, 2023). NSSI is prospectively associated with a range of adverse clinical outcomes, including rehospitalization (van Alphen et al., 2017), onset of mental disorders (Ose et al., 2021; Wilkinson et al., 2018), and future suicide attempts (Kiekens et al., 2018; Ribeiro et al., 2016). In addition, NSSI predicts negative psychosocial outcomes throughout young people's development such as difficulties in identity functioning (Gandhi et al., 2017), impaired emotion regulation abilities (Robinson et al., 2019), academic problems (Kiekens et al., 2016), interpersonal conflict (Waals et al., 2018), and heightened stigma resulting from visible scarring (Staniland et al., 2021). These outcomes are particularly pronounced among those who engage persistently and repetitively in NSSI (Kiekens et al., 2022; Steinhoff et al., 2021), underscoring the need for timely and effective interventions.

Despite growing recognition and clinical research efforts (Muehlenkamp and Tillotson, 2023), interventions for NSSI remain only modestly effective. A meta-analysis of randomized controlled trials found that conventional interventions over the past 50 years yield generally no significant differences or only small effect sizes regarding reduction of NSSI (Fox et al., 2020). However, interventions based on Cognitive Behavioral Therapy (CBT) and Dialectical Behavior Therapy (DBT) have recently shown promise through skills training and the development of alternative behavioral strategies (Linehan et al., 2015). Several recent programs based on CBT-DBT, including the Cutting Down Program (CDP; Kaess et al., 2020), Emotion Regulation Individual Therapy (ERITA; Bjureberg et al., 2018), and Treatment for Self-Injurious Behaviors (T-SIB; Andover et al., 2020) have further demonstrated efficacy in reducing NSSI among young people (Calvo et al., 2022).

The overall modest effectiveness of interventions to date may thus not reflect a treatment “ceiling” (Kleiman et al., 2022), but a mismatch between the predominantly static way in which assessment and intervention are delivered, typically at specific time points, and the inherently dynamic nature of NSSI (Kiekens et al., 2024), characterized by rapid within-person fluctuations. Recent research using Ecological Momentary Assessment (EMA; also referred to as Experience Sampling Method: a structured diary method that administers brief questionnaires multiple times per day via smartphone; Myin-Germeys and Kuppens, 2026) has demonstrated that NSSI-related cognitions (thoughts, urges, self-efficacy to resist NSSI) and behaviors fluctuate rapidly within individuals (Kiekens et al., 2024). Moreover, the transition from NSSI thoughts and urges to NSSI behavior typically occurs within 30 minutes (Fitzpatrick et al., 2020; Nock et al., 2009), and NSSI behaviors are more likely to occur during evening hours (Kiekens et al., 2024). Together, these findings highlight a critical mismatch: the static and retrospective nature of conventional assessments and interventions insufficiently allows us to provide care where and when NSSI cognitions and behaviors occur in daily life. Consequently, individuals are often left to manage acute distress outside the therapy room, where recalling and applying therapeutic skills can be difficult (Zieve et al., 2019). As such, there is a need for complementary approaches that align with the dynamic nature of NSSI.

Ecological Momentary Interventions (EMIs), which leverage EMA to deliver digital interventions via smartphone in individuals' everyday lives, may have clinical utility in addressing this gap by extending the reach of interventions beyond the therapy room (Beames et al., 2026). EMIs have been explored across several mental health conditions, including depression and psychosis (Folkersma et al., 2021; Bouws et al., 2025). Initial findings suggest that EMIs may hold promise for NSSI (Arshad et al., 2020), yet this remains an underexplored area, particularly regarding the perspectives of individuals who self-injure. Existing work highlights the importance of grounding EMIs in CBT and DBT, which provide individuals with adaptive emotion regulation skills to reduce NSSI behavior (Calvo et al., 2022; Melia et al., 2020). Simultaneously, recovery-oriented frameworks advocate a holistic approach that extends beyond merely reducing NSSI behavior, focusing instead on the broader regulation of vulnerability, realistic expectations for change, self-acceptance and self-compassion, and the promotion of mental health (Lewis and Hasking, 2021; da Cunha Lewin et al., 2023). A particularly promising advancement within EMIs is the development of just-in-time adaptive interventions (JITAIs; Wang and Miller, 2020). JITAIs aim to deliver the right type of support at the right moment by adapting intervention content and timing to an individual's changing risk and receptivity (Coppersmith et al., 2022; Nahum-Shani et al., 2018). However, before such interventions can be developed and implemented effectively for self-injury, it is essential to understand how treatment-seeking individuals and professionals perceive their potential utility and scope.

### 1.1 The present study

Using a qualitative interview design, we explored the perspectives of individuals who self-injure, after completing a four-week EMA period, and mental health professionals regarding: (1) the utility of EMA and EMI during treatment; (2) what to address, how, and when to intervene; and (3) the clinical deployment of an EMI/JITAI for NSSI, including how it could be used and integrated within the therapy process. These objectives were registered prior to analysis on the Open Science Framework (Luijsmans et al., 2024).

## 2 Method

### 2.1 Study design and data collection

The qualitative interviews were collected as part of the Detection of Acute rIsk of seLf-injurY (DAILY) Project (see protocol in Kiekens et al., 2023), a large-scale EMA study that includes 132 Dutch-speaking treatment-seeking individuals (aged 15 to 39 years) from 21 mental health services across the Belgian regions of Flanders, and their mental health professionals. Recruitment for this study began in June 2021 and concluded in August 2023. The data were collected during three phases: a baseline assessment (phase 1), 28 days of EMA during treatment followed by a clinical session between the participant and their mental health professional in which the EMA data were discussed, and a feedback survey (phase 2), as well as two follow-up surveys and an optional interview (phase 3; Kiekens et al., 2023). The clinical session was part of ongoing treatment and focused on discussing an individual's EMA report. Participants did not receive an EMI as part of the DAILY Project and this concept was introduced only during the interviews, which explored their perspectives on the potential use and design of EMIs (Kiekens et al., 2023). Previous empirical reports have all used quantitative EMA and survey data (Bonnier et al., 2025; Eggermont et al., 2026; Kiekens et al., 2024; Robillard et al., 2025). The DAILY Project was approved by the Ethics Committee Research UZ/KU Leuven (s64989), with informed consent obtained from all participants.

### 2.2 Participants who were interviewed

Following the 28-day EMA period, treatment-seeking individuals were invited to participate in an optional interview. Thirty of the 124 treatment-seeking individuals volunteered to participate and were interviewed (Table 1), together with all the participating mental health professionals. This resulted in 44 interviewees: 30 treatment-seeking individuals aged 18–39 years (*M* = 24.53, *SD* = 5.31), most of whom were women (80.0%) and did not have a college degree (70.0%), and the 14 mental health professionals treating the interviewed treatment-seeking individuals (all licensed clinical psychologists) aged 25–60 years (*M* = 28.64, *SD* = 10.82), most of whom were also women (78.6%). Among the professionals, 28.6% had worked with individuals who engage in NSSI for more than 20 years. The sociodemographic and clinical characteristics of the interviewed treatment-seeking individuals were comparable to those of the overall DAILY sample (Kiekens et al., 2024). Eligibility criteria, including criteria related to NSSI, are described in detail in the DAILY study protocol (Kiekens et al., 2023).

**Table 1:** Sociodemographic and clinical description of treatment-seeking individuals.

	Percentage (number) / Median (range)		Percentage (number) / Median (range)
**Age (18–39 years)**	24.53 (5.31)	**Past 12 Months NSSI Thoughts:**	
**Sex:**		< 5 times	0.0% (0)
Female	80.0% (24)	5–20 times	10.0% (3)
Male	20.0% (6)	21–50 times	6.7% (2)
**Sexual Orientation:**		51–100 times	30.0% (7)
Heterosexual	53.3% (16)	+100 times	53.3% (16)
Gay/lesbian	13.3% (4)	**Past 12 Months NSSI behavior:**	
Bisexual	33.3% (10)	< 5 times	3.3% (1)
**Current DSM-5 Mental Disorders:**		5–20 times	20.0% (6)
Major depressive disorder (last month)	76.7% (23)	21–50 times	26.6% (8)
Generalized anxiety disorder (past six months)	56.7% (17)	51–100 times	23.3% (7)
Post-traumatic stress disorder (last month)	50.0% (15)	+100 times	26.6% (8)
Panic disorder (last month)	50.0% (15)	**Past Month NSSI Thoughts:**	
Eating disorder (past three months)	43.3% (13)	1–4 times	3.3% (1)
Alcohol use disorder (past 12 months)	23.3% (7)	5–20 times	36.7% (11)
Other substance use disorder (past 12 months)	50.0% (15)	21–50 times	46.7% (14)
Any DSM-5 mental disorder	100.0% (30)	51–100 times	0.0% (0)
Number of DSM-5 mental disorders	5.0 (1–7)	+100 times	13.3% (4)
**Screen Borderline Personality Disorder:**	80.0% (24)	**Past Month NSSI Behavior**	93.3% (28)
**Mode of Care at Enrolment:**		**Past Month Times NSSI Behavior**	7 (0–90)
Inpatient care	80.0% (24)	**Required Medical Treatment for NSSI:**	
Outpatient care	0.0% (0)	No, it was not necessary	16.7% (5)
Hybrid care (combination)	20.0% (6)	Yes	80.0% (24)
**Lifetime NSSI Behavior:**		No, but it was necessary	3.3% (1)
5–20 times	3.4% (1)	**Uncontrollable urges present**	76.7% (23)
21–50 times	17.2% (5)		
51–100 times	24.1% (7)		
+100 times	55.2% (16)		
**Top Three Prevalent Methods:**			
Cut or carved skin	93.3% (28)		
Scratched skin	80.0% (24)		
Smashed hand/foot against wall or objects	76.7% (23)		
**Lifetime Number of Methods**	7.0 (3−11)		
**Age of Onset NSSI Behavior:**	14.5 (9–25)		
Childhood (11 years or younger)	6.7% (2)		
Adolescence (12–18 years)	73.3% (22)		
Emerging adulthood (19–29 years)	20.0% (6)		
Adulthood (30–39 years)	0.0% (0)		

### 2.3 Procedure of the interviews

Separate semi-structured interview guides were developed for treatment-seeking individuals and professionals (see Supplementary Material for Dutch and English versions). After an introduction, open-ended questions were used to explore: (1) perspectives on EMA and EMI utility during treatment, (2) what, how, and when to intervene in the risk process, and (3) practical use of an EMI/JITAI. Interviews with treatment-seeking individuals were conducted online, whereas those with mental health professionals were held on-site. Participation in the interviews was voluntary, and no compensation was provided.

All interviews were audio-recorded, transcribed verbatim without the use of AI, anonymized, and securely stored. The duration of the interviews ranged from 13 to 48 minutes (median = 23 minutes) for treatment-seeking individuals and from 14 to 43 minutes (median = 28 minutes) for professionals. All interviews were conducted by research staff (SS and NK) who were licensed clinical psychologists holding master or doctoral degrees.

### 2.4 Data analysis

Transcripts were analyzed using reflexive thematic analysis (Braun and Clarke, 2022), which enables exploration and interpretation of meaning, patterns, and depth of perspectives on the utility and scope of EMA and an EMI for NSSI, and recognizes the skills of the researcher as an analytic asset, rather than a source of bias (Braun and Clarke, 2021). We adopted an inductive, realist (essentialist) approach, considering participant's descriptions as meaningful and direct reports of their experiences, with codes and themes developed through in-depth engagement with the data (Braun and Clarke, 2022). First, ML listened to the audio recordings and read the transcripts multiple times to familiarize with the data, making notes and generating initial codes. Second, ML coded relevant or meaningful segments of data in two consecutive rounds using NVivo (Release 15.0.0 (12), 2024). Co-authors (LC, GK) independently coded three different transcripts, which were discussed and reflected upon before the second coding round. Third, initial themes were constructed based on the coded data and the overarching research questions, followed by refinement, fine-tuning with other co-authors, and writing-up the themes and analytic process, integrating and interpreting the data with theory. Once the themes for the treatment-seeking individuals were developed, the professionals' transcripts were analyzed. An audit trail of the analytic process is available in the Supplementary Materials.

## 3 Results

Table 2 shows the three main themes that were identified, integrating shared and contrasting views of treatment-seeking individuals and professionals. Each theme is presented through its key components and illustrated by participant's quotations (Table 3) clarifying their perspectives on the utility and scope of EMA/EMIs. Based on these perspectives, a new model for future application of an EMI for NSSI was developed (Fig. 1).

**Table 2:** The themes resulting from the reflexive thematic analysis with key components.

Themes	Key components
Theme 1: Extending therapeutic support into daily life	1. EMA/EMIs can provide a sense of not being alone. 2. EMA/EMIs may enhance awareness, reflection, and insight. 3. EMA/EMIs should be easily accessible.
Theme 2: Intervening depends on the moment and person: Risk and interventions as dynamic	1. EMIs should target changing momentary needs tied to varying levels of risk. 2. EMIs should be tailored to the person, not just the risk state.
Theme 3: Navigating deployment of EMIs	1. EMIs are useful when integrated within blended care and guided by shared decision-making. 2. EMIs should support the therapeutic alliance and promote autonomy. 3. EMIs/JITAIs should combine user- and system-initiated interventions.

**Table 3:** Illustrative quotes from participants for each theme and the conceptual intervention model.

Themes	Quote number	Illustrative quotes
Theme 1: Extending therapeutic support into daily life	1	TSI^a^ 5: *“I think that receiving a notification at a moment when you are not feeling well, you are reminded that you are not completely alone, because something is thinking about you. And I think that for some people that can already be very meaningful, even if it is something that small.”*
	2	TSI 19: *“You learn to pause more on things from therapy and the things you then have to do in the app, you reflect on them a bit more, so indirectly it actually helps … You are thinking about it and reflecting on it, so it does have an impact on your therapy because it makes you pause and reflect more.”*
Theme 2: Intervening depends on the moment and person: Risk and interventions as dynamic	3	Professional 8: *“They need a personalized list that we can review together. When someone is moving in the yellow zone, we consider what they can do at that stage, while all destructive behaviors are assigned to the red zone … People can then identify, for example, that listening to music might still be effective in the yellow zone but no longer in red, unless it is a different type of music. The same applies to physical activity. For instance, someone might manage a short walk with the dog in the yellow zone, while in red they might prefer something more intense such as boxing.”*
	4	TSI 27: *“Maybe the app shouldn't just suggest ‘look for distraction’, because that term has been used so much in treatment that it no longer feels meaningful to me, and I think others feel the same. Distraction is also very general, so it would help if the app suggested specific options that fit the person. Distraction can mean anything from having a coffee to going for a walk or listening to music, but in difficult moments it is hard to think of options when you are stuck in your thoughts. It would help to have a kind of support plan in the app with a clear list tailored to you like walking the dog, or doing something creative, or taking a bath. Having these options written out would provide guidance, and it would be important to be able to adjust the list, because people change over time and the plan should remain flexible.”*
A Needs-Based Dynamic Risk and Intervention Model for NSSI		
*Resilient (green) state: Mental health promotion interventions*	5	TSI 18: *“It is always just really helpful to not forget to take a moment for yourself, to invest in yourself in all the busyness. It might be good to maybe have a reminder to do something for yourself every day, even if it is something small. Something that helps to maintain the positive. Or like naming three positive things, or even just one thing you did this morning.”* … TSI 8: *“By pausing for a moment during the day to notice how strong the urge is, you can sometimes do something preventative when you notice the urge is already there but still not very strong. If you would not pause to notice it, you might not even realize it. And then you can do something about it.”*
	6	Professional 2: *“One of the basic things, but many of our patients no longer have, is a basic structure. Eating properly, getting enough sleep, those are things that are often neglected. People often get stuck there. When someone is readmitted, it's often one of the things that went wrong. We just see that regulating your emotions doesn't work if your basics aren't in order. If you're starving yourself or if you haven't slept all night, of course your emotions will be much more intense during the day. So I think looking at the basics first is really important.”*
	7	TSI 8: *“I think a very important aspect is motivation, wanting to stop. So maybe this is something you could work on at a moment when your head is a bit clearer by writing down why you want to stop, what the benefits could be, and also the long term benefits. And then maybe you could see these reasons again at moments when you do feel like hurting yourself. I think that might help.”*
*Emerging (yellow) risk state: Recognize vulnerability interventions*	8	TSI 21: *“It feels like we can't really sense what happens in between. It seems to go from zero to a hundred, and it's actually too late. They say that something always comes before it, we just don't see it.”*
	9	Professional 1: *“It may be valuable to help patients distinguish between primary and secondary emotions as patients often feel something very primary and quite quickly this is followed by secondary responses that are often destructive. If they could be asked to pause for a moment and think about what was there before the secondary response, this could be a good tool. Very often the secondary emotions get questioned, especially with our borderline population. Things like dysfunctional schemas such as self-punishment, feeling undeserving, or fear of losing control, that fear often arises because something else was felt first. So it would be useful to explore that, not only focusing on the present moment, but also on what was felt a few minutes earlier.”*
*High (red) risk state: Behavioral interruption interventions*	10	Professional 3: *“What's the problem? You say, ‘intense sadness’, and then the app could ask ‘what have you already tried?’ And if you say, ‘I've already tried to distract myself’, then the app could respond ‘I have a few other suggestions, have you tried using cold water? Or doing a breathing exercise?’ Something like that. Or, ‘I have those change-oriented suggestions, but at the same time, sadness is also an important human emotion, so maybe it could also suggest making space for that feeling?’, something like that.”*
	11	TSI 14: *“For me, activities such as exercising really help. So an intervention like that would work for me, as it would allow me to mark in the app that I actually did it. And it would be nice if the app kept track of that in the background, so you can see that you managed to complete the interventions.*”
	12	TSI 19: *“When something happens, like crisis behavior or even a crisis experience without actual behavior, we have to make a chain analysis … Even though I have grown to really dislike doing these after all this time, it does help. It starts with what the problematic or crisis behavior was, depending on how severe it was. The second point is the triggering event, what set it off? And the third point is the vulnerability factors, what made you vulnerable? Then you move on to the chains, where you really have to write everything down about what happened and how you experienced it, not the entire day but specifically that situation. At the end you then describe what harm it caused for yourself and for others, what consequences it had for me and my environment, and what harm I caused myself.*
Theme 3: Navigating deployment of EMIs	13	TSI 21: *“An app like this really should be almost linked to the therapy process, because otherwise, just a general app in the app store, that's not going to work. But with a personal layer, with follow-up attached to it, then it could actually be helpful.”*
	14	Professional 5: *“It is the patient and the clinician who decide together on the goals of the therapy at the start. The therapist can then make suggestions, like ‘if you want to reach those goals, I would recommend trying these things,’ and the patient needs to have the freedom to say, ‘I would rather not do that’.”*
	15	Professional 4: *“At the same time, we also expect people to really learn to assess for themselves what is difficult for them, why it is difficult, and to think in advance about what to do when it becomes hard and how they are going to handle it. I would be a bit concerned that this part, taking responsibility for themselves, learning to recognize signals, might get lost if there's an app that just says, ‘do this’.”*
	16	TSI 16: *“I think it would be most helpful when you already start to feel unwell and in those moments it would be good if the app could offer a reminder like ‘hey, I am still here to support’ or something like that.”*

a TSI: Treatment-seeking individual.

**Fig. 1:**
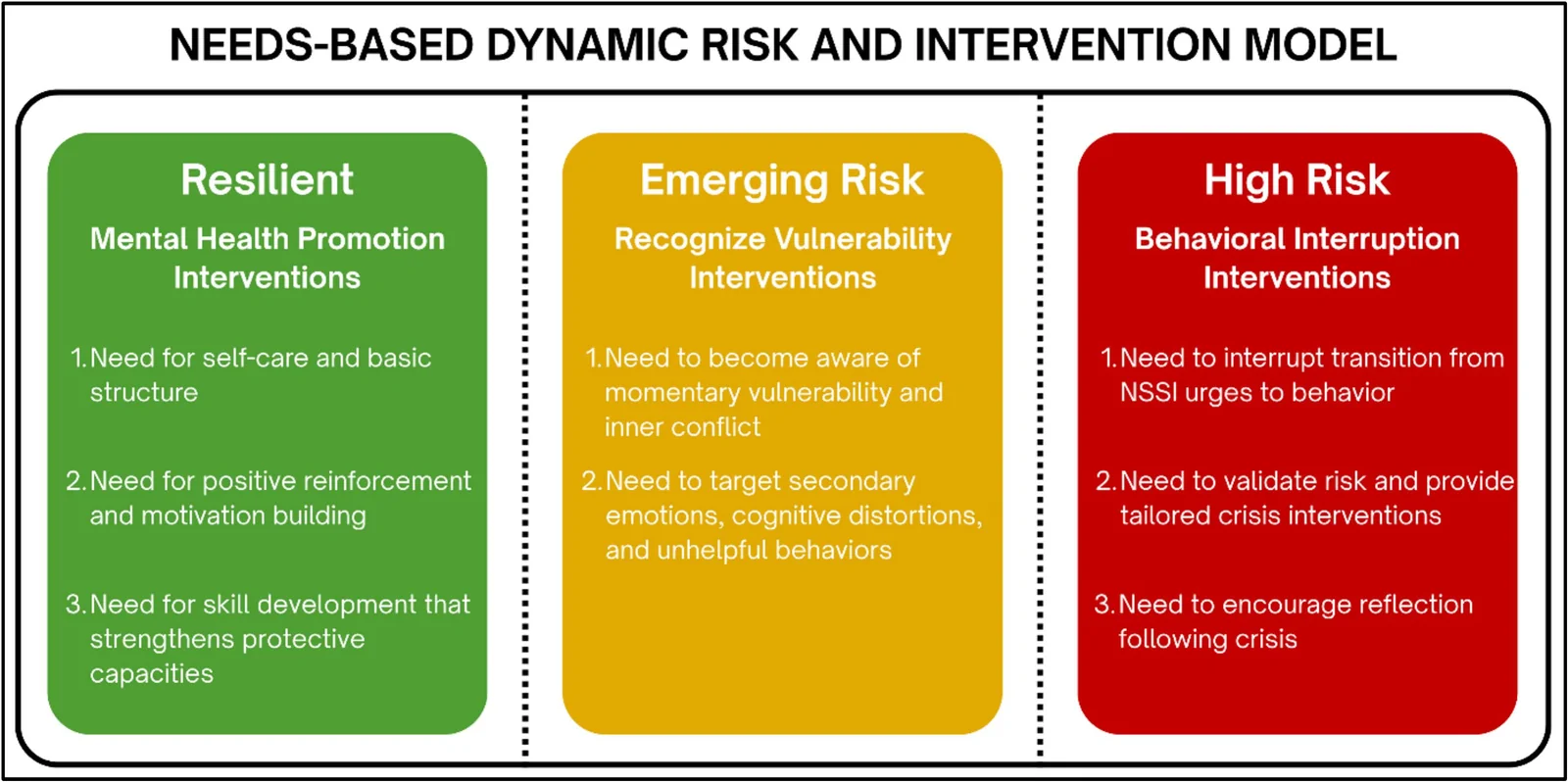
An overview of the Needs-Based Dynamic Risk and Intervention Model which resulted from the reflexive thematic analysis.

### 3.1 Theme 1: extending therapeutic support into daily life

The first theme captured how treatment-seeking individuals and professionals viewed EMA/EMIs as accessible digital tools that could extend therapeutic support into daily life. First, they described how features such as EMA prompts or EMI-based check-in exercises could create a sense of not being alone during difficult moments (Table 3, Quote 1). Participants mentioned several types of supportive content that an EMI could deliver during periods of heightened distress (e.g., *“I know you are struggling, but you are not alone”*), for example encouraging users to reflect on their thoughts and urges or to reach out to others for social support, either digitally or in-person, when conventional resources are unavailable. Second, EMA and EMIs were perceived as facilitating awareness, reflection, and skill use in daily life. Treatment-seeking individuals described EMA notifications as helpful for learning to pause and gain insight into emotional, cognitive and behavioral patterns over time (Table 3, Quote 2). Professionals echoed these views, describing an EMI as complementing clinical work by supporting patients in recognizing patterns of escalation and transferring skills learned during treatment into real-world contexts. They also emphasized that EMA could facilitate a more accessible, accurate, and continuous understanding of a patient's well-being, particularly when staff shortages limit the possibility for face-to-face assessments. Finally, individuals and professionals emphasized how the immediate accessibility of these digital tools would naturally align with modern lifestyles in which most people own a smartphone. At the same time, treatment-seeking individuals stressed that accessing an EMI should be easy and straightforward, especially when experiencing higher levels of distress and NSSI urges, as even a few additional steps on their smartphone could create barriers.

### 3.2 Theme 2: intervening depends on the moment and person: risk and interventions as dynamic

The second theme reflected that an EMI must address changing momentary and personal needs dynamically. Participants shared an understanding that what an EMI should target changes on what that person needs, with these momentary needs closely tied to the level of risk, which calls for different kinds of support. To illustrate this, participants often used a traffic-light analogy, describing green (resilient), yellow (emerging risk), and red (high risk) states, each associated with different needs and interventions an EMI should address. This helped illustrate how the type of support that feels helpful can shift depending on how someone is feeling and their state of risk. For example, going for a walk or listening to music might be useful distraction strategies when someone starts to feel worse (yellow) but may no longer be effective when someone is in a crisis (red). As one professional explained, certain activities may work in a “yellow zone,” but not in a “red zone”, where more immediate and intensive support is required (Table 3, Quote 3).

Simultaneously, participants stressed that it is not only about matching interventions of an EMI to momentary needs and risk, but also about fitting them to the person. Strategies that are effective for one individual may not be effective for another, and the utility of specific strategies is likely to evolve throughout the course of therapy as new skills and coping strategies are developed. It was mentioned that general interventions such as “find distraction” can feel too vague, especially in difficult moments when it is hard to think of concrete options. Therefore, treatment-seeking individuals expressed a preference for having a personalized list of strategies that are helpful to them, such as a safety plan, which can be adjusted over time as their needs and skills change (Table 3, Quote 4). These accounts indicated that an EMI not only requires a range of intervention components and options, but also sensitivity to changing circumstances, as its usefulness depends on the individual's current level of risk, their needs, and the context in which it is delivered.

### 3.3 A Needs-Based Dynamic Risk and Intervention Model for NSSI

Drawing on these perspectives, a Needs-Based Dynamic Risk and Intervention Model for NSSI was formulated to help guide the future application and development of an EMI for NSSI (Fig. 1). The model reflects the shared view of participants that what should be targeted in daily life should vary depending on a person's current risk and needs and provides examples of potential interventions and their delivery characteristics (i.e., when, how, and under which conditions support should be provided). Across momentary risk states, the EMI should flexibly shift its goals from (1) promoting mental health and strengthening protective routines (“green” state), to (2) helping individuals recognize and respond to emerging vulnerability (“yellow” state), to (3) interrupting or de-escalating acute NSSI urges to prevent a transition from thoughts to behavior during a crisis (“red” state).

#### 3.3.1 Resilient (green) state: mental health promotion interventions

At a resilient state, individuals and professionals described a need for interventions that promote mental health, enhance motivation, and support the practice of skills aimed at preventing future vulnerability. Treatment-seeking individuals particularly emphasized a need for self-care practices, such as taking brief moments during the day to pause and slow down, as meaningful ways to check-in with themselves and reflect on their feelings and needs in the moment. However, they noted that they do not consistently engage in these practices by themselves. An EMI was therefore considered helpful in supporting and reinforcing these mental check-ins, facilitating their incorporation into daily routines. For example, participants described how an EMI that prompts brief pauses, such as meditation exercises, or that encourages attention to positive experiences, such as reflecting on something positive from the day and being grateful, was perceived as helpful to maintain a resilient state over time (Table 3, Quote 5). Similarly, professionals emphasized the need for maintaining a consistent daily basic structure to sustain this resilient state, noting that irregular sleep, poor nutrition, and inactivity can undermine emotion regulation and increase vulnerability. They suggested that an EMI could help detect changes in lifestyle routines (e.g., *“I noticed your sleep hasn't been great lately, could this be a sign that you're not feeling well?”*) and provide gentle prompts to support engagement in basic self-care activities (Table 3, Quote 6).

Both treatment-seeking individuals and professionals described a need for positive reinforcement within the EMI to support motivation for change and engagement in therapy to remain resilient. Encouraging messages (e.g., *“you are doing well”*) were described to acknowledge daily efforts and progress, while reinforcing treatment goals. In doing so, acknowledging positive moments and one's own responsibility in creating them was seen as supporting motivation to change. Treatment-seeking individuals described a need to reflect on reasons for change, including the benefits and challenges of reducing NSSI, and to imagine a future self without self-injury (Table 3, Quote 7). Finally, resilient states were considered particularly suitable for meeting needs related to skills practice to enhance self-awareness, emotion regulation, and emotional expression, thereby strengthening protective capacities and helping to prevent future risk-escalation.

#### 3.3.2 Emerging (yellow) risk state: recognize vulnerability interventions

When individuals transition into an emerging state of risk, intervention needs shift from prevention and consolidation to recognizing increased vulnerability, calming down, and regulating emotions, thoughts, and behaviors. Professionals emphasized that recognizing heightened momentary vulnerability is an essential step in treatment, as this awareness is a prerequisite for timely adjustment and reducing self-injury and, where present, other self-damaging behaviors (e.g. disordered eating). This was relevant given that individuals described that they often experience an abrupt shift from low to high risk, without being able to detect this intermediate state of increased vulnerability (Table 3, Quote 8). As a result, learning to reflect on this emerging risk state was described as a goal during treatment. To meet needs related to detecting and responding to increased vulnerability, professionals highlighted the importance of slowing down and becoming aware of internal experiences, something an EMI could support with. This included recognizing an inner conflict between holding on to familiar habits (e.g., NSSI behavior when feeling emotionally overwhelmed) and striving for change (e.g., engaging in alternative non-harmful behaviors) during moments of heightened vulnerability. This inner conflict was characterized as involving increased tension such as physical agitation, emotional reactivity, and intensified negative thoughts. An EMI could support users by prompting real-time recognition of this inner conflict and facilitating reflection on its cognitive, emotional, and physiological characteristics during increased vulnerability.

According to professionals, an EMI could further support this process of recognizing and responding to increased vulnerability by integrating acceptance- and change-oriented strategies (i.e., dialectic position). This could be achieved by cultivating self-compassion and validating emotions and cognitions, while simultaneously encouraging small adjustments. Professionals described how an EMI could support awareness of emotional experiences during moments of increased vulnerability by prompting users to pause and attend to what they were feeling before self-criticism occurred (Table 3, Quote 9). As distress increases, self-critical thoughts and cognitive distortions may amplify vulnerability for NSSI. An EMI could support individuals in creating distance from these thoughts by encouraging reflection and perspective-taking, for example by questioning how they perceive themselves and others in that moment (e.g., *“is this thought actually true?”*). Finally, professionals described how an EMI could offer a range of personalized strategies aligned with therapy goals to help individuals adjust their actions during increased vulnerability.

#### 3.3.3 High (red) risk state: behavioral interruption interventions

At a high-risk state, participants described needs related to interruption of the transition from NSSI urges to behavior, validation of risk, and support following NSSI relapse or successful resistance. First, both treatment-seeking individuals and professionals emphasized that interventions at this stage should focus on preventing NSSI urges from turning into NSSI behaviors. Interventions need to occur before individuals become overwhelmed or lose their ability to reflect on alternative responses. To meet this need, professionals envisioned an EMI as valuable when it can communicate in an interactive and responsive way, like a coach providing support in a moment of crisis. By engaging users in a brief coach-like dialogue, an EMI could validate the difficulty of managing moments of intense distress, while also reminding that NSSI urges are temporary (e.g., “*This is difficult to bear right now, but you've managed it before, and this will pass too*”) and suggesting tailored interventions to help manage the crisis in a self-caring rather than self-damaging way (Table 3, Quote 10).

Complementing this, individuals described that interventions should combine validation with clear and concrete guidance during moments of high risk. Feeling validated and not feeling alone was perceived as meaningful in combination with specific strategies. The EMI could prompt immediate action aimed at distraction, suggesting personalized options such as doing household chores, listening to music, going outside, or seeking social support. In addition, an EMI could display an individuals' crisis plan to remind users of practiced skills, and encourage seeking support from significant others or a professional. DBT-skills, such as STOP, which helps to pause before acting on an impulse, assess the situation, and choose a non-harmful response, and CALM, which involves using cold, slowing breathing, relaxing muscles, and engaging in intense physical movement, were described as potentially helpful in reducing emotional overstimulation and regaining control. Follow-up and feedback on whether a strategy was used and experienced as helpful were also considered essential components at this stage (Table 3, Quote 11).

Finally, participants described the need to reflect following a crisis, to find out why they could not resist NSSI or what helped them resist the urge to engage in NSSI. An EMI could support individuals in reflecting on what happened through a guided chain analysis, helping them to identify triggering events and the sequence of thoughts, emotions, and behaviors that led to the crisis. This process was described as helping to target avoidance and support greater awareness of the behavioral chain, thereby contributing to longer-term change by, for example, intervening earlier in the chain (Table 3, Quote 12).

### 3.4 Theme 3: navigating deployment of EMIs

A final theme captured how treatment-seeking individuals and professionals envisioned the deployment of EMIs. Rather than a stand-alone tool, the EMI was described as most valuable when integrated into existing treatment, complementing face-to-face therapy. Professionals emphasized that EMA- and EMI-derived information could be flexibly incorporated depending on the treatment setting (e.g., individual, group-based, in- or outpatient) to support attunement to individuals' daily experiences and monitoring of change. Treatment-seeking individuals highlighted that this blended-care integration may help maintain a sense of personal connection and prevent the EMI from feeling like “*just another app*” (Table 3, Quote 13). Professionals considered an EMI particularly useful during early stages of therapy, where they could provide additional support, facilitate anticipation of risk, and reinforce skill use between sessions. Within this blended context, participants stressed that decisions about EMI use should be made through shared decision-making, rather than routinely implemented, considering preferences, readiness for change, and technology familiarity of both treatment-seeking individuals and professionals (Table 3, Quote 14).

Second, professionals stressed that the deployment of an EMI should enhance rather than replace the therapeutic alliance and process. While an EMI was seen as helpful in facilitating dialogue and providing information on an individual's daily life, professionals expressed concern that the EMI might replace in-vivo contact and help-seeking, which are vital skills to practice in the therapeutic relationship. Professionals stressed that this human connection – a living mind thinking along, empathizing, and attuning – remains the core of therapy and cannot be replaced by technology. If not carefully integrated, individuals may come to rely on the EMI to notice emotions or guide responses, rather than developing these skills in therapy (Table 3, Quote 15). Finally, when introduced to the concept of a JITAI, participants viewed it as promising to detect early signs of escalation and provide timely prompts to increase awareness or encourage adaptive coping during moments of increased risk (Table 3, Quote 16). However, participants mentioned that the most helpful JITAI should combine user- and system-initiated interventions. This combination would preserve user-control, foster autonomy and reassurance that support is always available, while ensuring timely support at vulnerable moments when initiating adaptive behaviors becomes challenging, thereby lowering the threshold for help-seeking. Participants envisioned a JITAI as evolving alongside the therapeutic process, offering more system-initiated support during early stages and periods of vulnerability while gradually stepping back as individuals develop confidence and self-reliance.

## 4 Discussion

This study explored the perspectives of individuals who self-injure and professionals regarding the utility and scope of EMA and EMIs during treatment. Three findings warrant further discussion. First, EMA and an EMI were envisioned as extensions of therapy by enhancing self-awareness, insight, and skill use in daily life. Second, an EMI should offer interventions that can dynamically adapt to changing needs in response to fluctuating risk for NSSI, which led to the formulation of a Needs-Based Dynamic Risk and Intervention Model. Finally, participants believed that the deployment of an EMI should be based on shared decision making to safeguard the therapeutic relationship and patients' autonomy. Each of these findings are discussed.

First, participants experienced EMA and EMIs as tools that can extend therapeutic support into daily life. Individuals who self-injure may experience difficulties with emotional awareness, negative thinking patterns, and social interactions (Houben et al., 2017; Hughes et al., 2019; Kiekens et al., 2020; Taylor et al., 2018). Our findings add to a growing body of literature suggesting that repeated self-assessment through EMA can enhance awareness of emotional, cognitive, and behavioral chains in real-world contexts (Bonnier et al., 2025; Bouws et al., 2025; de Thurah et al., 2025; Gromatsky et al., 2022; Kiekens et al., 2020), allowing treatment-seeking individuals to deploy alternative coping strategies and increase self-efficacy to resist NSSI (Hasking et al., 2017). Professionals similarly valued the information generated through EMA, as it enables a more continuous and ecologically-valid understanding of a patient's behavioral pattern (Bos et al., 2019; Bos et al., 2020) that can be integrated into ongoing treatment. While shame and stigma surrounding NSSI often hinder disclosure and help-seeking (Andersson et al., 2024; Mitten et al., 2016; Rosenrot and Lewis, 2020; Wang et al., 2023), treatment-seeking individuals could envision an EMI serving as easily accessible support that may reduce feelings of isolation, lower barriers to help-seeking, and facilitate communication within therapy. These findings support the potential of EMA and an EMI to better align interventions with the dynamic nature of NSSI during treatment (Glenn and Kleiman, 2025; Kiekens et al., 2024).

Second, the present study provides a specific account of how an EMI could address changing momentary needs in response to dynamic risk fluctuations in daily life, rather than being developed as a one-size-fits-all tool. This was illustrated by participants through a traffic-light analogy, which informed the development of a Needs-Based Dynamic Risk and Intervention Model in which intervention goals shift from promoting mental health (“green”), to supporting recognition of increased vulnerability (“yellow”), to interrupting the transition from NSSI urges to behavior (“red”). While there has been debate about the utility of risk screening at the population-level (Coppersmith et al., 2024; Hawton and Pirkis, 2024), this typically conceptualizes risk as subject-static (i.e., someone's risk ‘status’ is elevated compared to others) which does not capture intrapersonal short-term experiences and momentary changes in needs over time. Treating risk as a dynamic momentary process may therefore enable more need-sensitive support (Pisani et al., 2022), extending beyond crisis responses to maintaining well-being and preventing vulnerability in daily life. This aligns with person-centered recovery frameworks for NSSI, emphasizing that recovery is a dynamic, nonlinear and multifaceted process (Lewis and Hasking, 2021; da Cunha Lewin et al., 2023), and suggests that an EMI should be able to adapt to changing needs, addressing NSSI-related emotions, cognitions, and behaviors and the regulation of vulnerability more broadly (e.g., fostering self-acceptance and self-care; Lewis and Hasking, 2021; da Cunha Lewin et al., 2023). This flexibility is characteristic of a JITAI, which uses information about individual's changing needs and momentary states to adapt the timing and/or content of intervention delivery (Nahum-Shani et al., 2018).

Building on the Needs-Based Dynamic Risk and Intervention Model, a future EMI for self-injury, potentially operationalized as a JITAI, may draw on evidence-based strategies in a dynamic risk-sensitive manner by operationalizing interventions based on existing NSSI treatments (Andover et al., 2020; Bjureberg et al., 2018; Kaess et al., 2020; Linehan et al., 2015). This perspective is also reflected in emerging digital interventions for NSSI, such as the Kalmer app, which incorporates CBT and DBT components to address changing emotional states (Julià et al., 2026). Our findings complement these developments by highlighting the potential value of matching intervention content to dynamic states of resilience, vulnerability, and crisis and the needs associated with these states. At a resilient state (“green”), interventions may focus on maintaining well-being and strengthening motivation for change (Gander et al., 2016; Neuhaus et al., 2022), by incorporating positive psychology (e.g., savoring or gratitude; Fiß et al., 2026; Schreuder et al., 2026; Seligman et al., 2006), motivational interviewing techniques (e.g., developing discrepancy, rolling with resistance, supporting self-efficacy; Kress and Hoffman, 2008) and value-based reflections from Acceptance and Commitment Therapy (ACT; Engle and Follette, 2018; Hayes et al., 2012). Given the established link between identity functioning and NSSI (Eggermont et al., 2026; Gandhi et al., 2017), interventions may also focus on self-perception and interpersonal understanding (Grove and Smith, 2022). Beyond promoting well-being, this phase may be used to practice and consolidate skills that can be applied during periods of increased risk, which may include impulse control strategies (Gratz and Gunderson, 2006; Bjureberg et al., 2017), DBT and ACT skills (Linehan, 2025; Hayes et al., 2012), and strategies aimed at reducing negative emotions and cognitions (e.g., rumination, hopelessness, low self-esteem; Dehghan Manshadi et al., 2025), and applying alternative behaviors. In addition, reframing self-critical beliefs could be supported through compassion-focused interventions (Gilbert, 2009). Emotions could be addressed by imagery of emotions as a wave, meaning they are temporary and will pass (Gilbert, 2009; Rauschenberg et al., 2021).

When an individual's risk increases in-the-moment (“yellow”), interventions may begin to focus on increasing awareness of emerging vulnerability and supporting timely adjustment of emotional, cognitive, and behavioral responses. Participants described how this involves slowing down and recognizing internal conflict between the desire to change and the pull of habitual responses, which aligns with DBT-informed processes such as activating the Wise Mind and related skills (Linehan, 2025). Although the interviews did not yield specific decision rules for detecting emerging risk, participants described several potential markers of emerging vulnerability (e.g., physical agitation, emotional reactivity, intensified negative thoughts, and self-critical thinking). Awareness of these experiences, including differentiating between primary and secondary emotions (Linehan, 2025), may help individuals recognize increased vulnerability and identify when to apply strategies to regulate distress. In this phase, an EMI could draw on a range of strategies, including impulse control techniques (Gratz and Gunderson, 2006; Gratz et al., 2014; Bjureberg et al., 2017; Linehan, 2025), ACT-based approaches targeting avoidance of internal experiences (e.g., Creative Hopelessness; Hayes et al., 2012), and skills practiced at lower risk states. As risk escalates to a crisis state (“red”), intervention priorities shift toward interrupting the transition from NSSI urges to behavior. Participants emphasized the importance of timely, brief, and concrete interventions that combine validation of distress with actionable distracting strategies (e.g., DBT skills, Urge Surfing; Hayes et al., 2012; Linehan, 2025). In addition, safety planning (Großmann et al., 2025; Oexle et al., 2021) with the combination of enhancing coping strategies and support-seeking have already shown feasibility in the context of suicide prevention (Schatten et al., 2025). Finally, the period following a crisis was indicated to represent an important opportunity for reflection about why it was difficult to prevent NSSI behavior or what helped them from engaging in NSSI (Bjureberg et al., 2017).

Third, our findings highlight the importance of how EMIs are deployed within therapeutic settings. Participants viewed an EMI for self-injury as most valuable when embedded in a blended-care approach that complements face-to-face therapy. This perspective aligns with prior research and guidelines for digital interventions targeting suicidal behavior in youth (Bauer and Moessner, 2012; Bailey et al., 2024; Schueller et al., 2017). Without therapist involvement, individuals questioned the usefulness of an EMI, while professionals expressed concerns that excessive reliance on technology could undermine the development of skills that rely on human connection. Consequently, it is crucial for EMIs to find a balance in providing support in daily life while maintaining the therapeutic relationship as the core component of care (Andersson et al., 2024). One way to achieve this may be to combine user- and system-initiated interventions, providing system-initiated support during early stages of treatment and emerging vulnerability, while gradually stepping back as individuals progress through treatment. In this respect, participants valued the potential of a JITAI that provides system-initiated signals when vulnerability increases, while also emphasizing the importance of being able to initiate support independently as a source of agency (Simonsson et al., 2021). Consistent with this, engagement with interventions appears to depend on the perceived match with individuals' needs (Elmer et al., 2025) and shared-decision making (de Thurah et al., 2025), highlighting the importance of momentary adjustability and personalization (Großmann et al., 2025; Leppin et al., 2025). Together, these findings emphasize the need for personalization and co-design of a future EMI for NSSI to safeguard the therapeutic alliance, support autonomy of users, and tailor decision rules to capture dynamic states of resilience and risk to address individuals' needs.

### 4.1 Limitations

The findings of this qualitative study should be interpreted considering limitations. First, although the treatment-seeking individuals were selected from geographically diverse treatment units across Flanders in Belgium, the extent to which these findings can be interpreted within other non-clinical settings cannot be guaranteed and should be studied. Second, individuals who are not in treatment or differ in clinical characteristics may hold different beliefs about the blended-care application of EMIs, as these factors may shape perceived needs. Moreover, voluntary recruitment may have favored individuals more willing to participate in research. Third, given that all professionals were psychologists and most individuals were treated within a DBT-oriented framework, the prominence of concepts such as chain analysis and crisis interventions may partly reflect their experiences. Future research could examine the applicability and perceived value of these concepts across a broader range of therapeutic orientations and treatment settings. Finally, participants' familiarity with repeated self-monitoring after completing a 28-day EMA protocol may have influenced their expectations regarding the potential utility and design of an EMI.

### 4.2 Implications and future research

The present findings suggest that an EMI for NSSI should flexibly adapt intervention goals across resilient and risk states, shifting from awareness and regulation to interruption and post-crisis learning, which may hold clinical implications and future research recommendations. First, participants envisioned an EMI/JITAI as a dynamic tool that evolves alongside therapy, offering more intensive support during early stages and periods of emerging vulnerability and gradually reducing support as individuals develop more autonomy. Therefore, future research should examine how EMIs/JITAIs can be designed to adjust levels of support over time during treatment. Second, while the present study recruited individuals who self-injured, individuals may engage in several self-damaging behaviors (e.g., disordered eating, suicidal behavior; Ammerman et al., 2025; Kiekens and Claes, 2020). The present findings are specific to NSSI, and whether the proposed Needs-Based Dynamic Risk and Intervention Model may also be applicable to other self-damaging behaviors should be investigated in future research.

Third, future research should empirically validate the proposed dynamic assessment and intervention model by examining whether the qualitatively distinct resilient and risk states identified by participants can be represented as hidden dynamic states that fluctuate within individuals over time. Specifically, multilevel hidden Markov models (Aarts and Haslbeck, 2026) may help identify the latent structure of these states, characterize individual differences in state occupancy and transition dynamics, and determine the factors that predict movement between states in daily life. Such work could provide an empirical basis for developing JITAIs that adapt the therapeutic goals and intervention components to individuals' momentary needs and dynamic resilient and risk profiles (Mondal et al., in preparation). Furthermore, the intervention options identified for different states should be developed and tested through co-design and pilot studies with individuals with lived experience and professionals to optimize their relevance, acceptability, and responsiveness to changing needs. Building on these developments, micro-randomized trials (Klasnja et al., 2015) could be used to iteratively optimize adaptive intervention components and decision rules, thereby identifying which interventions are most effective for whom, under which dynamic states of resilience and risk, and in which contexts of daily life.

## 5 Conclusion

This is the first qualitative investigation to explore the perspectives of treatment-seeking individuals who self-injure and professionals on the role of EMA and EMIs in the treatment of NSSI. The findings suggest that these digital assessment and intervention tools have the potential to extend therapeutic support beyond the therapy room, enhance self-awareness, and facilitate timely responses to changing needs in daily life. Participants highlighted that effective support should adapt not only to fluctuations in risk but also to the changing goals and needs associated with different dynamic states of resilience, vulnerability, and crisis. A Needs-Based Dynamic Risk and Intervention Model was proposed that provides a framework for the development of a personalized and adaptive EMI/JITAI for individuals that self-injure and seek support. Future research should focus on empirically validating this model, and co-designing and optimizing intervention components that support individuals across dynamic states of risk and recovery in the context of blended care.

## Declaration of Generative AI and AI-assisted technologies in the writing process

During the preparation of this work the author(s) used ChatGPT (Open AI) and Microsoft Copilot to enhance grammar and readability of the manuscript without altering scientific content. After using these tools, the author(s) reviewed and edited the content as needed and take full responsibility for the content of the published article.

## Funding sources

This work was supported by the 10.13039/501100003130Research Foundation Flanders [12ZZM21N, GK; and 1182926N, ML]; and the support from a 10.13039/501100004040KU Leuven C1 grant [C16/23/011].

## Declaration of competing interest

The authors declare that they have no known competing financial interests or personal relationships that could have appeared to influence the work reported in this paper.
