# Supplementary material for: The perceived utility and scope of ecological momentary assessment and interventions for non-suicidal self-injury: A needs-based dynamic risk perspective
Source: Internet Interv. 2026 Oct 24;46:101014. doi: 10.1016/j.invent.2026.101014 (PMC13720625; doi:10.1016/j.invent.2026.101014)
Supplement: Supplementary file 1 [file mmc1.docx]

The Perceived Utility and Scope of Ecological Momentary Assessment and Interventions for Non-Suicidal Self-Injury: A Needs-Based Dynamic Risk Perspective

Luijsmans Mirthe, Claes Laurence, Tatnell Ruth, Houben Marlies, Vaessen Thomas, Smeets Tom, Myin-Germeys Inez, & Kiekens Glenn

The Research Audit Trail

Based on templates by Marian Carcary (2020; DOI: 10.34190/JBRM.18.2.008) and McLeod (2024; DOI: 10.13140/RG.2.2.13969.95842)

**Research problem identification and proposal development***:* Non-suicidal self-injury (NSSI; i.e., the intentional injury of one’s own body tissue without suicidal intent) is a major mental health problem characterized by rapid within-person fluctuations in NSSI thoughts and urges. Yet, existing treatments largely rely on retrospective and static assessment and intervention approaches, which are unable to capture the dynamic nature of NSSI. Ecological Momentary Assessment (EMA) and Ecological Momentary Interventions (EMIs) may deliver support dynamically beyond the therapy room, but their application to NSSI remains underexplored.

**Research aims and objectives***:* This qualitative study explored the perspectives of individuals with lived experience of self-injury and mental health professionals. The initial four research questions were described in the post-registration on the Open Science Framework (Luijsmans et al., 2024; <https://osf.io/9ef6q>). Eventually, the second and third research question were combined, resulting in these final three research questions: (1) the utility of EMA and EMIs during treatment, (2) what to address, how, and when to intervene, and (3) the clinical deployment of EMIs for NSSI.

**Research framework***:* The interviews were collected as part of the DAILY Project (Kiekens et al., 2023), a large-scale EMA study including 125 individuals seeking treatment (aged 15-39 years) and 14 mental health professionals. The interview transcripts were analyzed using reflexive thematic analysis (TA; Braun & Clarke, 2022), which allows for the exploration and interpretation of meaning, patterns, and depth within the perspectives of the interviewees on the utility and scope of EMA/EMIs for NSSI. Reflexive TA also recognizes the skills of the researcher as an analytic asset rather than bias (Braun & Clarke, 2021). We adopted a realist (essentialist) approach, considering participant’s descriptions as meaningful and direct reports of their experiences. Codes and themes were developed through in-depth engagement with the data.

**Sample selection***:* Participation in the interviews was voluntarily after the 28-day EMA period. Of the 125 individuals seeking treatment, 30 volunteered to participate in the interviews. Additionally, all 14 mental health professionals voluntarily participated.

**Data analysis process***:*

*Familiarization:* Familiarization was conducted on paper and notes were written in the margins of each transcript together with a summary at the end of each transcript. The first author conducted two rounds of familiarization of the 44 transcripts.

*Coding for individuals seeking treatment:* The coding process for individuals seeking treatment consisted of two coding rounds. In addition to coding on paper, NVivo was used to create a clear overview of the codes and code descriptions, with a new project or backup for each coding round. In a first round, the first author inductively coded the 30 transcripts of individuals seeking treatment, starting with the first transcript and working toward the 30^th^. Code names were created inductively, based on meaningful segments of text within the transcripts. Whenever new information was considered meaningful, either a new code was created or the first author reflected on whether this new information could fit under an already existing code. Codes were structured under the four initial research questions to create an easy overview of the codes (i.e., ‘Perspectives (RQ1)’, ‘Focus (RQ2)’, ‘How & When (RQ3)’, ‘JITAI (RQ4)’. An additional ‘Other’ category was created for meaningful segments that did not fall under any research question (e.g., information on stigma, current treatment, or other apps that individuals seeking treatment mentioned). The first coding round resulted in 164 codes. After finishing the first coding round for the 30 transcripts of individuals seeking treatment, the first author shared three different transcripts of these 30 with the second and last author who each coded their three transcripts independently. Afterwards, the first, second, and last author came together to discuss their codes and reflected on the content of the transcripts. Based on these reflections and her experiences during the first coding round, the first author revisited her coding book and created a first overall structure consisting of codes that seem to work around a central organizing concept, creating initial themes under each research question. This was followed by a second, more deductive coding round of the 30 transcripts of individuals seeking treatment starting at transcript 15 and ending at transcript 14 to disrupt assumptions formed during the first coding round and to re-engage with the data more reflexively and openly. During this second coding round new codes could still be created. The second coding round resulted in 242 codes.

*Initial theme construction for individuals seeking treatment:* To assure that the themes resulting from individuals seeking treatment would not be influenced by those of mental health professionals, initial themes were created solely based on the codes of individuals seeking treatment. All codes were printed out and cut into small pieces to create puzzle pieces, independent from the original research question ‘category’ they belonged to in NVivo, and start initial theme construction on a large table. These initial themes were revised over three rounds after reflexive discussions among the first, second and seventh author. Pictures of the coding puzzle table were taken during each round and notes were taken with descriptions and thoughts for each theme.

*Coding for mental health professionals:* The coding process for mental health professionals consisted of one coding round due to time constraints. Again, in addition to coding on paper, NVivo was used to create a codebook with a separate project and backup for the transcripts of the mental health professionals. The first author inductively coded the 14 transcripts of the mental health professionals, starting with the first transcript and ending with the 14^th^. Code names were created inductively, based on meaningful segments of text within the transcripts. Whenever new information was considered meaningful, either a new code was created or the first author reflected on whether this new information could fit under an already existing code. Codes were again structured under the four initial research question and an additional ‘Other’ category to create an overview of the codes. This coding round resulted in 350 codes.

*Initial theme construction for mental health professionals:* Initial themes were created solely based on the codes of mental health professionals in one theme construction round. All codes were again printed out (in a different color than the codes of individuals seeking treatment) and cut into small pieces to create a puzzle. Picture of the coding puzzle for mental health professionals were taken together with notes of descriptions and thoughts for each theme of the first author.

*Combined initial theme construction:* After consolidation of the mental health professionals, the coding puzzles of the individuals seeking treatment was placed on the table next to the coding puzzle of the mental health professionals. Starting from this broad overview of all codes and looking back at quotes, four initial themes were constructed by the first, second, seventh and final author.

*Initial writing:* These four themes were named, defined, and written out by the first author in combination with meaningful quotes to gain a deep and thorough understanding of the initial themes.

*Refining, defining, and naming themes:* In combination with the writing of the paper, the first and final author reflected together over multiple rounds on the themes. Starting from each research question they wanted to answer, they reflected on each theme and their key components while taking into account relevant quotes from the data. This resulted in five themes with their key components and an initial conceptual idea of the Needs-Based Dynamic Risk and Intervention Model.

*Writing:* During the writing of the results section of the manuscript, the first, second, and final author reflected and discussed the themes and the model over 16 versions. The themes were refined and combined, resulting in three themes. The first, second, third, and final author reflected on and refined the Needs-Based Dynamic Risk and Intervention Model based on the codes and quotes within the data that provided specific input for this model. The manuscript was revised based on feedback of all co-authors.

**Reflexivity statement:** Thematic analysis never happens in a “theoretical vacuum” (Braun & Clarke, 2006) and the researchers reflected on and were aware of how their own backgrounds, experiences, and perspectives might have influenced interpretation of the data. The first author is a PhD researcher at the Center for Contextual Psychiatry at KU Leuven, studying perspectives of individuals with lived experience of NSSI and is a clinical psychologist. After familiarization of the transcripts, she noticed that many participants received Dialectical Behavior Therapy (DBT) or worked within this psychotherapeutic framework. To better understand the theoretical concepts within the transcripts, the first author observed how DBT works within a ward of the UPC Kortenberg psychiatric hospital in Belgium for six months. Throughout the study, the first author maintained a reflexive journal to document evolving interpretations, responses to the data, decisions regarding coding, and reflections on how prior knowledge may have influenced analytic choices. For example, the researcher noted moments where prior knowledge of the importance of the therapeutic alliance strongly aligned with participant accounts, prompting further reflection to avoid digital tools replacing the therapeutic alliance. Theme development involved iterative engagement with the data, repeated revisiting of quotes, and critical reflection on how analytic decisions were made. Discussions with co-authors also contributed to critical reflection of interpretations and helped challenge assumptions underpinning the analysis.
