# Supplementary material for: The perceived utility and scope of ecological momentary assessment and interventions for non-suicidal self-injury: A needs-based dynamic risk perspective
Source: Internet Interv. 2026 Oct 24;46:101014. doi: 10.1016/j.invent.2026.101014 (PMC13720625; doi:10.1016/j.invent.2026.101014)
Supplement: Supplementary file 2 [file mmc2.docx]

**DAILY project**

**The interview guide for participants & clinicians**

**PARTICIPANTS INTERVIEW**

Dear participant,

Welcome to this interview!

We are here because you indicated in the feedback questionnaire that you are willing to provide your perspective and additional feedback on self-monitoring and the scope of future digital interventions. Thank you so much for helping us out with this!

The interview will take approximately 30 minutes. It is important that you answer the questions as honestly as possible. You may not have an answer ready to all questions, but you can take your time to think about it and try to formulate a response. Your opinion matters, so please note that there are no right or wrong answers.

The audio of this conversation will be recorded. Your answers and the recording will be treated confidentially. If you have any questions or experience any difficulties answering the questions, be sure to let us know.

Let's get started!

Experience of self-monitoring as a digital tool in therapy

1. Can you tell me what it was like to use the m-Path app for a month?
2. Do you feel that self-monitoring has changed your behavior, thinking, or feelings toward therapy in any way?
3. Do you think digital self-monitoring could add value to your recovery and therapy? Why/why not?

Utility and scope of future digital interventions

4. To support adolescents and (young) adults like yourself, we would like to be able to provide digital interventions (via smartphone) when people indicate they are having a difficult time. Do you think this is a relevant objective? Why/why not?
5. What would you like a digital intervention to be able to help/support you with in daily life?
6. Suppose you would have to choose one specific theme or topic that a digital intervention could help you with; what would that be?
7. How would a digital intervention be able to help you with this? Do you have any suggestions for this?
8. Are there certain things that a digital intervention would not be able to help you with?

*Thank you for your answers so far. We now would like to talk to you about interventions that could support people like you in their recovery from self-injury or other self-damaging behaviors (such as binge eating, intentional vomiting, and suicidal thoughts).*

9. Would a digital intervention be able to help you resist the urge to self-injure yourself in everyday life? Why/why not?
10. How would a digital intervention be able to help you with this? Do you have any suggestions for this?
11. Could the same intervention also be helpful in the recovery process of other self-damaging behaviors (such as binge eating, intentional vomiting, and suicidal thoughts)? Why/why not?

- If not: What should an intervention directed towards [indicated topic] focus on?

12. To what extent would you like to have control over the availability of an intervention (i.e., do you want to be able to choose whether you receive an intervention at an (emotionally) difficult moment)?
13. This will be reflected in your answers when you're having a difficult time. Now suppose that the researchers could succeed in developing a *smart* tool that can detect these changes in your mood or emotional state, and 'warn' you in time (e.g., before a strong urge to injure yourself arises) and offer an intervention. Would you like to use this tool in therapy? Why/why not?
14. Are there any important aspects or issues that have not yet been discussed but that we should consider when developing digital interventions?

Thank you for participating in this interview.

**CLINICIANS INTERVIEW**

Dear colleague,

Welcome to this interview!

In this interview, we will ask you about your perspective on the utility of self-monitoring for your clients and the further development of digital interventions.

The interview will take approximately 30 minutes. It is important that you answer the questions as honestly as possible. You may not have an answer ready to all questions, but you can take your time to think about it and try to formulate a response. Your opinion matters, so there are no right or wrong answers.

The audio of this conversation will be recorded. Your answers and the recording will be treated confidentially. If you have any questions or experience problems answering the questions, be sure to let us know.

Let's get started!

Experience of self-monitoring as a digital tool in therapy

1. What was your initial attitude toward self-monitoring?
2. How receptive were your clients toward the self-monitoring tool?
3. Did you find the questions in the app relevant and personal enough? Were there any relevant questions missing in the feedback report?
4. What effect do you think the self-monitoring had on your clients?
5. How relevant is it to implement digital self-monitoring in mental health care?
6. Do you think using self-monitoring can be valuable in treating self-injury and other self-damaging behaviors (e.g., binge eating, intentional vomiting, suicidal thoughts)? Why/why not?

- If so: can you indicate how this could be of value?
- If not: can you indicate why not?

How did you (and your team) experience the safety protocol?

Utility and scope of future digital interventions

7. To support adolescents and (young) adults, we would like to be able to provide digital interventions (via smartphone) when people indicate they are having a difficult time. Do you think this is a relevant objective? Why/why not?
8. What would you like a digital intervention to help/support your clients with in daily life?
9. Suppose you would have to choose one specific theme or topic with which a digital intervention could help your clients; what would it be?
10. What should this intervention look like? Do you have any suggestions for this?

*Thank you for your answers so far. We now would like to talk to you about interventions that could support clients in therapy and their recovery from self-injury or other self-damaging behaviors (such as binge eating, intentional vomiting, and suicidal thoughts).*

11. Would a digital intervention be able to help your clients in resisting the urge to self-injure in everyday life? Why/why not?
12. How would a digital intervention be able to help them with this? Do you have any suggestions for this?
13. Could the same intervention also be helpful in the recovery process of other self-damaging behaviors (such as binge eating, intentional vomiting, and suicidal thoughts)? Why/why not?

- If not: What should an intervention directed towards [indicated topic] focus on?

14. This will be reflected in your client’s answers when they are having a difficult time. Now suppose that the researchers could succeed in developing a smart tool that can detect these changes in the client's mood or emotional state (e.g., before a strong urge to self-injure manifests ) and offer an intervention. Would you like to use this tool in your therapy? Why/why not?
15. Are there any important aspects or issues that have not yet been discussed but that we should consider when developing digital interventions?

Thank you for participating in this interview.
