# Supplementary material for: The perceived utility and scope of ecological momentary assessment and interventions for non-suicidal self-injury: A needs-based dynamic risk perspective
Source: Internet Interv. 2026 Oct 24;46:101014. doi: 10.1016/j.invent.2026.101014 (PMC13720625; doi:10.1016/j.invent.2026.101014)
Supplement: Supplementary file 3 [file mmc3.docx]

**DAILY project**

**Interview gids cliënten en behandelaars**

Interview met cliënten

Beste cliënt,

Welkom bij dit interview! Wij zijn hier aanwezig omdat je in de vorige vragenlijst hebt aangegeven bereid te zijn om extra feedback te geven rond het gebruik van een digitale tool. Alvast bedankt dat je ons hierbij wil helpen!

In dit interview zal je mening gevraagd worden over zelfmonitoring en de ontwikkeling van digitale interventies.

Het interview duurt ongeveer 30 minuten. Het is belangrijk dat je de vragen **zo eerlijk mogelijk** beantwoordt. Het kan zijn dat je bij sommige vragen niet meteen een antwoord klaar hebt, maar denk er even over na en probeer toch een antwoord te formuleren. Het gaat erom wat jij vindt, er zijn dus geen goede of foute antwoorden.

De audio van dit gesprek zal opgenomen worden. Er wordt vertrouwelijk omgegaan met jouw antwoorden en de opname. Indien je vragen hebt of problemen ondervindt bij het beantwoorden van de vragen, geef dit dan zeker aan.

Laten we van start gaan!

Ervaring van zelfmonitoring als een digitale tool in therapie

1. Kan je me vertellen hoe het was om de m-Path app voor een maand te gebruiken?
2. Heb je het gevoel dat zelfmonitoring jouw gedrag, denken of handelen, of je gevoel bij de therapie heeft veranderd?
3. Denk je dat digitale zelfmonitoring op zich een meerwaarde kan zijn voor je herstel en therapie? Waarom wel/niet?

Perspectief aangaande het potentieel en de de inhoud van toekomstige digitale interventies

4. Om adolescenten en (jong)volwassenen zoals jou te ondersteunen, zouden we in de toekomst graag (via de smartphone) interventies kunnen aanbieden op het moment dat men aangeeft het moeilijk te hebben. Vind je dit een relevante doelstelling? Waarom wel/niet?
5. Waar zou jij willen dat een interventie je mee zou kunnen helpen/ondersteunen in het dagelijkse leven?
6. Stel dat je één specifiek thema of onderwerp zou moeten kiezen waar de interventie je bij helpt, wat zou dat dan zijn?
7. Hoe zou een digitale interventie jou hierbij kunnen helpen? Heb je hier suggesties voor?
8. Zijn er zaken waar digitale interventies jou niet mee zouden kunnen helpen?

*Dankjewel voor je antwoorden tot dusver. We zouden het nu graag even met je hebben over interventies die mensen zoals jou zouden kunnen ondersteunen in hun herstel van zelfverwonding of andere gedragingen (bv. eetbuien, opzettelijk overgeven, zelfdodingsgedachten).*

9. Zou een digitale interventie je kunnen helpen om de drang naar zelfverwonding te weerstaan in het dagelijkse leven? Waarom wel/niet?
10. Hoe zou een digitale interventie jou hierbij kunnen helpen? Heb je hier suggesties voor?
11. Zou eenzelfde interventie ook helpend zijn in het herstelproces van andere gedragingen (bv. eetbuien, opzettelijk overgeven, zelfdodingsgedachten)? Waarom wel/niet?

- Indien niet: Waar zou een interventie voor [aangegeven onderwerp] dan wel op moeten focussen?

12. In welke mate zou je controle willen hebben over de beschikbaarheid van een interventie (bv. wil je kunnen kiezen of je een interventie ontvangt op een (emotioneel) moeilijk moment)?
13. Wanneer het minder goed met je gaat, dan gaan we dat zien in je antwoorden. Stel nu dat onderzoekers erin slagen om een *slimme tool* te ontwikkelen die deze veranderingen kan detecteren en jou tijdig (d.w.z. vooraleer een sterke drang ontstaat) kan ‘waarschuwen’ en een interventie kan aanbieden, zou je dan van die tool gebruik willen maken in therapie? Waarom wel/niet?

Afronding

14. Zijn er volgens jou belangrijke aspecten of zaken die nog niet aan bod zijn gekomen, maar waar we wel rekening mee moeten houden in de ontwikkeling van digitale interventies?

Dankjewel voor dit interview.

Interview met behandelaars

Beste collega,

Welkom bij dit interview!

In dit interview zal je mening gevraagd worden over zelfmonitoring en de ontwikkeling van digitale interventies.

Het interview duurt ongeveer 30 minuten. Het is belangrijk dat je de vragen **zo eerlijk mogelijk** beantwoordt. Het kan zijn dat je bij sommige vragen niet meteen een antwoord klaar hebt, maar denk er even over na en probeer toch een antwoord te formuleren. Het gaat erom wat jij vindt, er zijn dus geen goede of foute antwoorden.

De audio van dit gesprek zal opgenomen worden. Er wordt vertrouwelijk omgegaan met jouw antwoorden en de opname. Indien je vragen hebt of problemen ondervindt bij het beantwoorden van de vragen, geef dit dan zeker aan.

Laten we van start gaan!

Ervaring van zelfmonitoring als een digitale tool in therapie

1. Wat was jouw initiële attitude ten opzichte van zelfmonitoring?
2. Hoe ontvankelijk waren jouw cliënten met betrekking tot het gebruik van de zelfmonitoring tool?
3. Vond je de vragen in de app relevant en persoonlijk genoeg? Waren er relevante vragen die volgens jou ontbraken?
4. Wat was volgens jou het effect van het zelfmonitoring op jouw cliënten?
5. Hoe relevant is het volgens jou om een digitaal zelfmonitoring te implementeren in de geestelijke gezondheidszorg?
6. Denk je dat het gebruik van zelfmonitoring een meerwaarde kan hebben in de behandeling van zelfverwonding en andere zelfbeschadigende gedragingen (bv. eetbuiten, opzettelijk overgeven, zelfdodingsgedachten)?

- Ja: Kan je aangeven hoe?
- Nee: Kan je aangeven waarom niet?

Hoe heeft u als behandelaar/team het veiligheidsprotocol ervaren?

Perspectief aangaande het potentieel en de de inhoud van toekomstige digitale interventies

7. Om adolescenten en (jong)volwassenen te ondersteunen, zouden we in de toekomst graag (via de smartphone) interventies kunnen aanbieden op het moment dat men aangeeft het moeilijk te hebben. Vind je dit een relevante doelstelling? Waarom wel/niet?
8. Waar zou jij willen dat een interventie cliënten je mee zou kunnen helpen/ondersteunen in het dagelijkse leven?
9. Stel dat je één specifiek thema of onderwerp zou moeten kiezen waar een interventie cliënten in zou kunnen ondersteunen, wat zou dat dan zijn?
10. Hoe zou een dergelijke interventie eruit moeten zien? Heb je hier suggesties voor?

*Dankjewel voor je antwoorden tot dusver. We zouden het nu graag even met je hebben over digitale interventies die cliënten (die in therapie zijn) zouden kunnen ondersteunen in het herstel van zelfverwonding of andere zelfbeschadigende gedragingen (bv. eetbuien, opzettelijk overgeven, zelfdodingsgedachten) in het dagelijkse leven*.

11. Zou een digitale interventie cliënten kunnen helpen om de drang naar zelfverwonding te weerstaan in het dagelijkse leven? Waarom wel/niet?
12. Hoe zou een digitale interventie hen hierbij kunnen helpen? Heb je hier suggesties voor?
13. Zou eenzelfde interventie ook helpend zijn in het herstelproces van andere zelfbeschadigende gedragingen (bv. eetbuien, opzettelijk overgeven, zelfdodingsgedachten)? Waarom wel/niet?

- Indien niet: Waar zou een interventie voor [aangegeven onderwerp] dan wel op moeten focussen?

14. Wanneer het minder goed gaat met je cliënt, dan gaan we dat zien in zijn/haar antwoorden. Stel nu dat onderzoekers erin slagen om een slimme tool te ontwikkelen die deze veranderingen kan detecteren en jouw cliënt tijdig (d.w.z. vooraleer een sterke drang ontstaat) een interventie kan aanbieden, zou je dan van die tool gebruik willen maken in je therapie? Waarom wel/niet?

Afronding

15. Zijn er volgens jou belangrijke aspecten of zaken die nog niet aan bod zijn gekomen, maar waar we wel rekening mee moeten houden in de ontwikkeling van digitale interventies?

Dankjewel voor dit interview.
